# Factors predicting the outcome of customised foot orthoses in patients with rheumatoid arthritis: a prospective cohort study

**DOI:** 10.1186/1757-1146-4-8

**Published:** 2011-02-10

**Authors:** Marike van der Leeden, Karin Fiedler, Annelies Jonkman, Rutger Dahmen, Leo D Roorda, Dirkjan van Schaardenburg, Joost Dekker

**Affiliations:** 1 Reade, Centre for Rehabilitation and Rheumatology (formerly Jan van Breemen Institute), Dept. of Rehabilitation Research, Amsterdam, Netherlands; 2 VU University Medical Centre, Dept. of Rehabilitation Medicine, EMGO Institute, Amsterdam, The Netherlands; 3 Reade, Centre for Rehabilitation and Rheumatology (formerly Jan van Breemen Institute), Dept. of Rheumatology, Amsterdam, The Netherlands; 4 VU University Medical Centre, Dept. of Rheumatology, Amsterdam, The Netherlands

## Abstract

**Background:**

Conservative management of foot problems in patients with rheumatoid arthritis (RA) may consist of the prescription of customised foot orthoses. Indications for foot orthoses are not clear and the effectiveness of the intervention is highly variable among patients. Knowledge on which patients benefit the most from foot orthoses can help to select patients eligible for this type of intervention. The objective of the present study was to determine clinical and demographic factors that predict the outcome of customised foot orthoses on pain and disability in patients with RA.

**Methods:**

A total of 135 RA patients who were supplied with customised foot orthoses were included in this prospective cohort study. Pain and disability were measured before and after the intervention period using a Numeric Rating Scale (NRS) for foot pain, the Foot Function Index (FFI), the Western Ontario and McMasters Universities Osteoarthritis Index (WOMAC) and a 10-meter walking test. The intervention period consisted of one or more appointments with the podiatrist during which the foot orthoses were customised.

Swollen foot joint count, foot deformity scores, forefoot peak pressure, disease duration, age, gender, body mass index and baseline values of the outcome measures were selected as potential factors predicting outcome. Multivariate linear regression analyses were performed to determine factors associated with change in pain and disability (at *P *< 0.05).

**Results:**

Disease duration was negatively associated with the change scores in NRS foot pain (*P *= 0.018), WOMAC pain (*P *= 0.001), FFI disability (*P *= 0.003) and WOMAC physical function (*P *= 0.002). Age was negatively associated with the change score in 10 meter walking time (*P *= 0.008). For all outcome measures baseline values were positively associated with the change scores (P < 0.001).

**Conclusions:**

Shorter disease duration predicted greater improvements in self-reported foot pain and disability, and younger age predicted greater improvements in walking time after intervention with foot orthoses. Also, higher baseline values of pain and disability predicted greater improvements. Referral for conservative management with foot orthoses in the early stage of RA seems important when aiming to achieve reduction in pain and improvement in daily activities.

## Background

Throughout the course of rheumatoid arthritis (RA) foot problems appear to be highly prevalent [1-5], most often causing pain during weight bearing activities such as standing, walking and running [4,6]. The primary management of RA-related foot problems is pharmacological, although conservative and/or surgical intervention may be indicated [7]. Conservative intervention may consist of foot orthoses in over-the-counter shoes or therapeutic footwear. Surgical intervention may be considered when conservative intervention is not satisfactory in reducing foot pain and consequent disability or when to attempt to obtain a better long term prognosis.

The effectiveness of foot orthoses has been shown in several randomized controlled trials [8-10]. However, indications for foot orthoses are not clear and the effectiveness of the intervention is highly variable among patients [11]. Knowledge on which patients benefit the most from foot orthoses can help to select patients eligible for this type of intervention. Studies investigating factors predicting the outcome of foot orthoses on pain and disability in RA are lacking to date. Several clinical and demographic factors may potentially predict the outcome. Hypotheses can be generated based on the literature and clinical experience.

Potential clinical predictors related to the foot include the presence and severity of disease activity in the foot joints, the presence and severity of structural foot deformities and the magnitude of pressure under the forefoot. Higher disease activity in the foot joints is expected to predict poorer outcome since disease activity and the associated pain are influenced by pharmacological treatment [7], rather than by foot orthoses. More severe structural foot deformities are also expected to predict poorer outcome since foot orthoses may be insufficient to accommodate to severe deformities. Moreover, the foot with severe deformities may not fit into over-the counter shoes [12]. High pressure under the forefoot is hypothesized to be associated with a better outcome, since foot orthoses in RA have been shown to reduce high forefoot pressure and consequently reduce pain and disability [13,14]. Another potential clinical predictor is disease duration. Shorter disease duration is expected to be associated with a better outcome since the consequences of the disease may be more amenable to intervention in an early disease stage than in a later disease stage when irreversible joint damage and deformities could have been developed [15-17].

Demographic factors, i.e. age and gender, and body mass index are also potential predictors of the outcome of foot orthoses. Older patients are expected to report poorer outcome since these patients experience additional age-related disabilities [18] which might be less responsive to an intervention with foot orthoses than RA-specific disabilities. Gender might also be a predictor of outcome. Some recent studies suggest that men with RA have better responses to pharmacological interventions than women with RA [19]. Based on these findings gender could potentially be a predictor although no evidence for gender differences in the outcome of conservative interventions in RA has been found. Body mass index (BMI) might be a predictor of outcome since overweight is related to co-morbidities, such as cardiovascular diseases [20], causing disabilities which are less likely to respond to an intervention with foot orthoses. Finally, baseline values of pain and disability are expected to be predictors of outcome on these measures. Patients with high baseline scores have more opportunity to improve than patients with already low baseline scores.

The purpose of the present study was to determine clinical and demographic factors that predict the outcome of customised foot orthoses on pain and disability in patients with RA.

## Methods

### Design

Patients of an outpatient centre for rehabilitation and rheumatology (Reade, formerly Jan van Breemen Instituut) in the Netherlands served as the study population for this prospective cohort study. Since 2006 patients with a referral to podiatry for arthritis-related foot complaints were assessed using standardized measurements. Patients have been measured at baseline (before the start of the intervention) and at final follow up (directly after the end of the intervention period). The intervention period consisted of one or more appointments with the podiatrist during which the foot problem was diagnosed and managed. Individual diagnosis and evaluation was the primary purpose of the measurements. In addition, data have been stored anonymously for evaluation of foot care on a group level. According to Dutch law, using data from routine health care for scientific purposes is permitted: no separate approval of the Human ethics board is required.

### Patients

For the present study the following criteria for inclusion were used: 1) RA diagnosed by a rheumatologist (according to the American Rheumatism Association 1987 revised criteria [21]), 2) referral to a podiatrist for RA-related foot complaints, 3) uni- or bilateral foot complaints, 4) intervention with customised foot orthoses and 4) older than 18 years of age. Exclusion criteria were: 1) any other medical condition that could explain the foot complaints, 2) inability to walk unassisted with or without walking aids and 3) inability to complete questionnaires (because of language or cognitive problems). The inclusion period was April 2006 to September 2009.

### Intervention

The foot orthoses were custom designed and manufactured by experienced podiatrists who were accustom to treating RA-related foot problems. There were three podiatrists involved. Most of the foot orthoses were constructed by using prefabricated orthoses. The half supplements were model U and made from ethylene vinyl acetate (EVA) shore 70 or Microcork shore 80 (Fisher Group bv, Meppel, the Netherlands). The full supplements were model Ergonomica and were made from EVA shore 60 (Fisher Group bv, Meppel, the Netherlands). The pre-fabricated orthoses were heated and moulded to the patient's foot while using the neutral suspension technique with handmade functional corrections [22]. Some orthoses were handmade using the Lavigne technique [22]). Various cover materials were used (leather, cloth material, spenco or no cover). For the majority of orthoses a cushioning material such as PPT or plastazoate was added at the forefoot before the cover was applied.

In addition to the prescription of foot orthoses, all patients were given advice regarding shoes. The general anatomy and characteristics of a shoe were explained and tailored advice was given which took into account the patient's individual needs. Characteristics such as shoe weight, shoe fitting including shoe depth and model (sandals, shoe or ankle high boots), heel height, sole stiffness and cushioning properties, malleability of the shoe upper and the fastening apparatus (laces, Velcro, buckle or zipper) were all considered. After giving the shoe advice patients were provided with a list of shoe shops selling appropriate shoes for patients with RA-related foot problems.

In some cases toe orthoses to address toe deformities were made. For example overlying toes were held in a better alignment with a silicone orthosis. Small shoe adaptations were performed when necessary, for example stretching or softening the leather upper at pressure points and different lacing techniques. In some cases, patients were referred to orthopaedic shoemakers for rocker bottoms or heel height corrections.

After receiving the foot orthoses patients returned for a review appointment with the podiatrist. During this appointment the foot orthoses were re-evaluated and could be altered when the effect was not satisfactory in reducing pain according to the patient. If necessary, follow up visits were arranged to further optimize the foot orthoses.

### Outcome measures

Pain and disability were measured at baseline (T0) and at final follow up (T1) using questionnaires and a walking test.

A Numeric Rating Scale (NRS) was used to assess foot pain during walking. Patients were asked to score the average amount of foot pain they had experienced during walking in the previous week using a NRS, where 0 indicates no pain and 10 indicates severe pain.

The Foot Function Index (FFI) was used to measure the impact of foot problems [23,24]. The scale consists of 23 items divided into 3 subscales: pain (9 items), disability (9 items) and activity restriction (5 items). For the present study a modified version of the FFI was used. The modified FFI uses a 5 point-scale for item response [24], where the original FFI uses a 100 mm VAS [23]. To calculate the subscale scores and the total score the item scores were summed, divided by the maximum possible sum of the item scores and then multiplied by 100. The scores range from 0 to 100; the higher the score the more pain, disability and activity restriction respectively. For the present study the subscales pain and disability were used as outcome measures.

The Western Ontario and McMasters Universities Osteoarthritis Index (WOMAC) was used to measure the impact of arthritis in the lower extremities. The WOMAC originally measures pain, joint stiffness and physical function as the impact of osteoarthritis in hips and knees [25,26]. However, the WOMAC has also been reported to be an appropriate measure of lower extremity related pain, joint stiffness and physical function in RA [27]. The WOMAC consists of 24 items divided into 3 subscales: pain (5 items), joint stiffness (2 items) and physical function (17 items). To calculate the subscale scores and the total score the item scores were summed, divided by the maximum possible sum of the item scores and then multiplied by 100. The scores range from 0 to 100; the higher the score, the more pain, stiffness and disability respectively. For the present study the subscales pain and physical function were used as outcome measures.

A 10-meter walking time test was used as a performance-based measure of physical function. The patients were instructed to walk 10 meters on a self-selected, comfortable walking pace while wearing their own shoes and -after the intervention- wearing the provided foot orthoses in their shoes. A walking aid was permitted during testing if patients used one for daily mobility. The time to walk 10 meters was recorded (in seconds). A comparable walking time test, that is the 50-foot walking time test, was found to be reliable, valid and responsive in patients with RA [28-30].

### Potential predictors

Potential factors predicting changes in pain and disability after the intervention with foot orthoses were measured at baseline (T0).

Gender, age (in years), body mass index (in kg/m^2^) and disease duration as time since medical diagnosis (in years) were recorded for each patient.

A swollen foot joint count was used to record disease activity in the foot joints. Only soft tissue swelling was accounted for [31]. Scoring was based on the absence (= 0) or presence (= 1) of joint swelling according to the podiatrist after palpation along the joint margins. Swelling of the interphalangeal joints, metatarsophalangeal (MTP) joints 1 to 5, tarsal complex, subtalar joint and talocrural joint was evaluated in both feet. Since no foot joint count has been studied for its validity or reliability, a foot joint count that was routinely used by the podiatrists of our centre was used as the most appropriate alternative. The number of swollen joints in both feet was summed (range 0-18).

The Structural Index score [32] was used to quantify forefoot and rearfoot deformities. Hallux valgus (absent = 0, present = 1), MTP subluxation (number 0-5), 5th MTP exostosis (absent = 0, present = 1), and claw/hammer toe deformities (number 0-5) were considered for the forefoot deformity score (range 0-12). Calcaneus valgus/varus angle (0-5° = 0; 6-10° = 1; 11-15° = 2; >15° = 3), ankle range of motion (46-60° = 0; 31-45° = 1; 15-30° = 2; <15° = 3) and pes planus/cavus deformitiy (absent = 0, present = 1) were considered for the rearfoot deformity score (range 0-7). The mean forefoot and rearfoot deformities scores of the right and left foot were used in the analyses.

Plantar pressure measurements were performed to determine peak pressure under the forefoot using an EMED^®^-nt (Novel Electronics, Novel gmbh, Munich, Germany) platform (4 sensors per cm^2^, sample frequency 50 Hz). The platform was mounted in the middle of a walkway of 5 meters. A two-step method of collecting plantar pressure measurements was used [33]. Data from both feet were collected and three correct measurements per foot were recorded. A measurement was rejected when the whole foot failed to be placed on the platform or when the patient was off balance according to the tester or to the patient. All patients started walking with their right foot. Pressure data were analysed with Novel-Ortho^® ^and Novel-Win^® ^software. A division mask (Novel-mask) identified the forefoot region. Peak pressure (PP) under the forefoot was calculated. The PP was determined by the highest pressure measured by a single sensor in the forefoot region. The mean forefoot PP (N/cm^2^) of the right and left foot was used in the analyses.

### Other measurements

The following data were used descriptively: site(s) of foot complaints as indicated by the patient (toes, forefoot, midfoot, subtalar joint and ankle; either uni- or bilateral), RA-related medication at T0 and T1 (including nonsteroid anti-inflammatory drugs (NSAIDs), disease modifying antirheumatic drugs (DMARDs) and biologicals (e.g. etanercept and infliximab)), number of appointments with the podiatrist, duration of the intervention period from intake to final follow up (in months) and type of podiatric intervention (type of foot orthosis and additional toe orthoses and/or shoe adaptations).

Foot- and disease-specific information was assessed and recorded by the podiatrists. Other measurements (i.e. questionnaires, walking test and plantar pressure measurements) were performed by an independent clinical research assistant. All assessors received training in taking the measurements in a standardised way.

### Statistical analyses

Descriptive statistics for baseline patient characteristics were calculated as means (SD), or medians (IQR) when data was not normally distributed. Differences between T0 and T1 scores on the outcome measures for pain and disability were analyzed with non-parametric Wilcoxon Signed Ranks Tests (with a significance level of *P *< 0.05) since the outcome measures were not normally distributed.

Multivariate linear regression analyses (backward selection) were performed to identify factors associated with change in pain and disability. A significance level of *P *< 0.05 was used to include a factor in the final model. The change scores in NRS foot pain, FFI pain, WOMAC pain, FFI disability, WOMAC physical function and 10-meter walking time were entered as dependent variables. A positive change score indicated an improvement in pain and disability. Potential predictors were entered as independent variables, i.e. swollen foot joint count, Structural Index score forefoot, Structural Index score rearfoot, forefoot peak pressure, disease duration, age, gender, BMI and baseline score of the outcome measure that was used as the dependent variable.

All analyses were performed using SPSS, version 18.0 (SPSS, Chicago, IL).

## Results

### Descriptive data

A total of 135 patients with RA were included in the present study. Nine patients had complete missing T1 data and were therefore excluded from the analyses. Baseline patient characteristics, which are simultaneously potential predictors of the outcome of foot orthoses, are shown in Table 1.

**Table 1:** Baseline patient characteristics/potential predictors (n = 126)

Gender, n	Females 95 Males 31
Mean (SD) age, years	54.9 (12.6)
Mean (SD) BMI, kg/m^2^	25.5 (4.2)
Median (IQR) disease duration, years	3.5 (1.0;10.0)
Median (IQR) swollen foot joint count, 0-18	0.0 (0.0;3.0)
Structural Index	
Median (IQR) forefoot score, 0-12	1.0 (0.0;3.0)
Median (IQR) rearfoot score, 0-7	1.0 (0.0;1.0)
Mean (SD) peak pressure forefoot, N/cm^2^	46.8 (17.2)

n = number, SD = standard deviation, IQR = interquartile range

The majority of the patients reported forefoot complaints (82.9%). NSAIDs were used by 24.6% of patients at T0 and 26.4% at T1, DMARDs were used by 87.3% of patients at T0 and 87.6% at T1 and biologicals were used by 16.9% of patients at T0 and 17.4% at T1.

The mean number of appointments with the podiatrist (including the intake) was 3.9 (SD 1.6). The mean duration of the intervention period was 3.6 months (SD 2.4 months).

Most of the foot orthoses that were supplied incorporated a combination of a deep heel cup for rearfoot stability or correction, a contoured medial arch for middle foot support and a metatarsal bar and dome for forefoot offloading (69.9%). In 16.5% of the patients only forefoot support/offloading was provided and in 13.6% a combination of midfoot and hindfoot support/correction was provided. Only a minority of patients were supplied with toe orthoses (7.3%) or small shoe adaptations (4.1%).

### Changes in pain and disability

Table 2 shows T0 and T1 scores on the outcome measures for pain and disability. Statistically significant improvements after the intervention with foot orthoses were found on all outcome measures *(P *< 0.001).

**Table 2:** Baseline (T0) and follow up (T1) scores for pain and disability measures

	T0 Mean (SD)	T1 Mean (SD)	Differences P-value
NRS foot pain, 0-10	4.9 (2.7)	3.5 (2.7)	< 0.001
FFI pain, 0-100	37.2 (20.6)	23.6 (18.3)	< 0.001
WOMAC pain, 0-100	32.8 (21.8)	23.0 (22.0)	< 0.001
FFI disability, 0-100	31.4 (22.5)	23.8 (22.5)	< 0.001
WOMAC physical function, 0-100	30.1 (22.5)	23.0 (21.7)	< 0.001
10-meter walking time, sec	9.6 (2.7)	8.9 (2.1)	< 0.001

### Factors predicting changes in pain and disability

The results of the multivariate linear regression analyses are shown in Table 3. Only variables that showed a statistically significant association (at *P *< 0.05) with change scores in the outcome measures were included in the final model. Disease duration was negatively associated with the change scores in NRS foot pain (*P *= 0.018), WOMAC pain (*P *= 0.001), FFI disability (*P *= 0.003) and WOMAC physical function (*P *= 0.002). Age was negatively associated with the change score in 10-meter walking time (*P *= 0.008). For all outcome measures baseline values were positively associated with the change scores (P < 0.001). The explained variance (R^2^) for the final models ranged from 19.2% to 46.1%.

**Table 3:** Results of multivariate linear regression analyses of change in pain and disability measures with statistically significant factors (*P *< 0.05)

	NRS foot pain (n = 109)	FFI pain (n = 107)	WOMAC pain (n = 108)	FFI disability (n = 107)	WOMAC physical function (n = 108)	Walking time (n = 103)
Variable	B (95% CI)	B (95% CI)	B (95% CI)	B (95% CI)	B (95% CI)	B (95% CI)
Disease duration	-0.064 (-0.118 to-0.011)		-0.679 (-1.087 to-0.270)	-0.584 (-0.968 to-0.201)	-0.588 (-0.962 to-0.214)	
Age						-0.026 (-0.044 to-0.007)
Baseline value	0.344 (0.192 to 0.495)	0.518 (0.371 to 0.664)	0.316 (0.173 to 0.459)	0.233 (0.111 to 0.355)	0.295 (0.171 to 0.418)	0.371 (0.291 to 0.452)
R^2^	0.196	0.325	0.235	0.192	0.240	0.461

Reduced patient numbers in the multivariate linear regression analyses were due to random missings in the outcome measures or selected predictors.

## Discussion

The present study was the first to identify factors predicting the outcome of customised foot orthoses on pain and disability in RA.

Shorter disease duration was found to be associated with a better outcome on self-reported measures of pain and disability. An explanation for this finding could be that the consequences of the disease may be more amenable for treatment in an earlier disease stage than in a later disease stage when irreversible joint damage and deformities could have been developed [15-17]. Our finding reflects our clinical experience that a podiatry referral in early RA is crucial when aiming to achieve reduction in pain, improvement in daily activities and prevention of further loss of foot function. This has been supported by a recently published editorial by Woodburn et al. [34], in which a 'window of opportunity' for best results for foot problem management by podiatry in early RA has been described. Management includes early detection of foot problems, targeted therapy, tight control of foot arthritis and disease monitoring. To make the most of this 'window of opportunity' innovative health care is required. One of the challenges facing combined pharmacological and non-pharmacological interventions is to target simultaneously both local inflammatory lesions and associated mechanically-based impairments [34]. These combined interventions need to be developed and evaluated to further optimize the management of foot problems in early RA.

Another finding of our study was that older age was associated with poorer outcome on a 10-meter walking time test. An explanation might be that older age is accompanied by age-related co-morbidities, which may hinder improvement in walking ability. Co-morbidities, especially cardiovascular diseases, have been reported to be frequent in RA and prevalence increases with older age [35].

As expected, higher baseline values of the outcome measures (indicating more pain or disability) were associated with more change on these measures after intervention with foot orthoses. This could be explained by a floor effect of the outcome measures which may have caused regression to the mean: patients with high baseline scores are able to improve more than patients with already low baseline scores who have less possibility to improve.

Contrary to our expectations we were not able to identify clinical factors that were related to the foot, i.e. swollen foot joint count, foot deformities and forefoot plantar pressure, as predictors of the outcome of foot orthoses. This finding could be explained by indication bias, which means that the referral for foot orthoses and the orthoses themselves were tailored to foot-related factors. For example, the Structural Index scores for foot deformities were relatively low, which might be due to the fact that patients with more severe deformities were referred for therapeutic footwear or orthopaedic consultations rather than for foot orthoses. To investigate whether foot-related factors predict the effect of foot orthoses another study design is needed, using a broader selection of patients (e.g. with more severe deformities) and an intervention with standard foot orthoses.

Statistically significant improvements in pain and disability after the intervention with customised foot orthoses were found. The clinical relevance of these results can be interpreted by comparing minimal important changes (MIC) that have been reported for the self-reported pain and disability measures that were used in the present study.

The mean change score on the NRS foot pain was -1.4 points. For patients with chronic musculoskeletal pain (including RA-related pain) a reduction of one point in NRS pain represented a MIC [36]. The NRS change score in our study may therefore be interpreted as a clinically relevant reduction in foot pain. Also, we found a mean change score in FFI pain of -14 points and a mean change score in FFI disability of -8 points. The only study reporting MICs for the FFI has been performed in patients with plantar fasciitis following conservative treatment [37]. MICs for FFI pain of -12 points and FFI disability of -7 points were found. Based on these MICs our results may be regarded as clinically relevant improvements in foot-related pain and disability. However, since plantar fasciitis and RA are significant different conditions it has to be considered that the MICs for these conditions may differ as well. Furthermore, a decrease of 30% from the baseline WOMAC pain score and a decrease of 24% from the baseline WOMAC physical function subscale was found in our study. In patients with osteoarthritis following a rehabilitation intervention the MIC for subscales of the WOMAC was 12% from baseline scores [38]. Based on the relatively large improvements found in our study these improvements in lower-extremity related pain and physical function may be regarded as clinically relevant.

Customising foot orthoses can be achieved using different methods. In our study the manufacturing of foot orthoses was variable depending on patient's specific needs and the education of the treating podiatrist. No standard prescription protocol was followed. To date, no evidence is available for which types of foot orthoses are most effective for RA [11]. Further research is needed for evidence-based prescription protocols for foot problems in RA.

We chose to use a hypothesis-oriented approach in the selection of potential predictive variables, as opposed to a more traditional explorative approach. The strength of a hypothesis-oriented approach is that all potential predictive factors are selected based on evidence in the literature, clinical experience and/or biological plausibility. In an explorative approach a large number of factors is tested univariately, and only factors with a statistically significant association with the outcome are selected for multivariate analysis [39]. In our study the number of potential predictive factors was limited which made it possible to enter all factors into the multivariate analysis without selection based on statistical methods.

There are several limitations to this study that warrant discussion. We only included factors measured at baseline, resulting in factors associated with the outcome on pain and disability which may give direction to a podiatry referral. However, factors regarding the intervention might also predict the outcome. An important intervention-related factor is the wearing time per day [11]. Although all patients indicated that they wore their foot orthoses we did not collect detailed information about the wearing time. Also, we did not record whether patients changed their footwear as part of the intervention. When the foot orthoses are being worn in inappropriate footwear the potential effect may be lost since a mutual influence of both the foot orthosis and the footwear on the outcome of the intervention is presumed [40]. Furthermore, we did not record other interventions than medication. Interventions such as exercise or advice concerning change in activity levels might have influence on foot-related pain and disability. We recommend to include these factors in future studies investigating predictors of outcome of foot orthoses.

Data concerning radiological damage of foot joints were lacking in our study. Instead, we obtained information about foot deformities, which can develop as a result of joint damage in combination with capsuloligamentous instability of the foot [41]. The advantage of deformities is that they are recorded by clinical examination, in contrast to radiological damage for which data are not available in all settings. Also a global disease activity measure, such as the frequently used DAS28 (Disease Activity Score including 28 joints) [42] was lacking. Instead, we recorded the use of medication during the intervention period as an indicator for disease activity. We concluded a rather stable disease during the intervention period since minimal changes in medication were recorded in the study population. Therefore, the improvements in pain and disability are likely to be attributed to the intervention with foot orthoses.

Our results support the evidence which has been provided from randomized controlled trials that foot orthoses are effective in patients with RA [8-10]. However, we did not include a control group for clinical course and therefore we are not able to conclude decisively that improvements in pain and disability were the result of the intervention with foot orthoses. Furthermore, we are not able to distinguish between predictors of the natural course of RA and predictors of treatment outcome. Identifying predictors of outcome in uncontrolled studies does not allow this distinction to be made. Whether or not a distinction in predictors exists can only be studied using data of both the experimental and the control group in a randomized controlled trial aimed at investigating the effectiveness of foot orthoses in RA.

## Conclusions

Shorter disease duration predicted greater improvements in self-reported foot pain and disability, and younger age predicted greater improvements in walking time after intervention with foot orthoses. Also, higher baseline values of pain and disability predicted greater improvements. Referral for conservative management with foot orthoses in the early stage of RA seems important when aiming to achieve reduction in pain and improvement in daily activities.

## Competing interests

The authors declare that they have no competing interests.

## Authors' contributions

ML coordinated the data collection, performed the statistical analysis and wrote the manuscript. KF collected data, participated in the design of the study and helped to draft the manuscript. AJ collected data and helped to draft the manuscript. RD, LR and DS participated in the design of the study and helped to draft the manuscript. JD participated in its design and coordination and helped to draft the manuscript. All authors read and approved the final manuscript.
